# Adenovirus vector-mediated single chain variable fragments target the nucleocapsid protein of porcine epidemic diarrhea virus and protect against viral infection in piglets

**DOI:** 10.3389/fimmu.2023.1058327

**Published:** 2023-01-24

**Authors:** Fengqing Wang, Qing Zhang, Fanqing Zhang, En Zhang, Mei Li, Shiwei Ma, Jianming Guo, Zhibiao Yang, Jianguo Zhu

**Affiliations:** ^1^ Shanghai Key Laboratory of Veterinary Biotechnology, School of Agriculture and Biology, Shanghai Jiao Tong University, Shanghai, China; ^2^ Department of Food Science and Technology, School of Agriculture and Biology, Shanghai Jiao Tong University, Shanghai, China; ^3^ State Key Laboratory of Advanced Optical Communication Systems and Networks, Key Laboratory for Laser Plasmas (Ministry of Education), School of Physics and Astronomy, Shanghai Jiao Tong University, Shanghai, China

**Keywords:** porcine epidemic diarrhea virus, nucleocapsid protein, single chain variable fragment, recombinant adenovirus, protective effects

## Abstract

Porcine epidemic diarrhea virus (PEDV) mainly infects the intestinal epithelial cells of pigs, causing porcine epidemic diarrhea (PED). In particular, the virus causes severe diarrhea, dehydration, and death in neonatal piglets. Maternal immunity effectively protects neonatal piglets from PEDV infection; however, maternal antibodies can only prevent PEDV attachment and entry into target cells, but have no effects on intracellular viruses. Intracellular antibodies targeting virus-encoded proteins are effective in preventing viral infection. We previously identified four single chain variable fragments (scFvs), ZW1-16, ZW3-21, ZW1-41, and ZW4-16, which specifically targeted the PEDV N protein and significantly inhibited PEDV replication and up-regulated interferon-λ1 (IFN-λ1) expression in host cells. In our current study, the four scFvs were subcloned into replication-defective adenovirus vectors to generate recombinant adenoviruses rAdV-ZW1-16, rAdV-ZW3-21, rAdV-ZW1-41, and rAdV-ZW4-16. ScFvs were successfully expressed in Human Embryonic Kidney 293 (HEK293) cells and intestinal porcine epithelial cell line J2 (IPEC-J2) and were biosafe for piglets as indicated by body temperature and weight, scFv excretion in feces, IFN-γ and interleukin-4 (IL-4) expression in jejunum, and pathological changes in porcine tissue after oral administration. Western blotting, immunofluorescence, and immunohistochemical analyses showed that scFvs were expressed in porcine jejunum. The prophylactic effects of rAdV-ZW, a cocktail of the four rAdV-scFvs, on piglet diarrhea caused by PEDV was investigated. Clinical symptoms in piglets orally challenged with PEDV, following a two-time treatment with rAdV-ZW, were significantly reduced when compared with PEDV-infected piglets treated with phosphate buffered saline (PBS) or rAdV-wild-type. Also, no death and jejunal lesions were observed. ScFv co-localization with the PEDV N protein *in vivo* was also observed. Next, the expression of pro-inflammatory serum cytokines such as tumor necrosis factor-α (TNF-α), IL-6, IL-8, IL-12, and IFN-λ was assessed by enzyme-linked immunosorbent assay (ELISA), which showed that scFvs significantly suppressed PEDV-induced pro-inflammatory cytokine expression and restored PEDV-inhibited IFN-λ expression. Therefore, our study supported a promising role for intracellular scFvs targeting the PEDV N protein to prevent and treat diarrhea in PEDV-infected piglets.

## Introduction

Porcine epidemic diarrhea (PED) is caused by porcine epidemic diarrhea virus (PEDV) and is one of the most lethal diseases in piglets; neonatal piglet mortality rates can reach 90%–100% and cause serious economic losses to the global pig breeding industry (1–4). Diseased piglets are clinically characterized by lethargy, loss of appetite, watery diarrhea, dehydration, and even death (1, 2). PED prevention measures have included enhanced biosecurity on pig farms and vaccinations (5, 6). However, more PEDV variant strains have been recently reported, and similarly, when PED outbreaks are caused by variant PEDV strains, traditional vaccines are largely ineffective in controlling infection and spread (7–9).

The neonatal piglet immune system is immature and takes 3 weeks to produce antibodies, therefore, maternal immunity is vital for protecting piglets against PEDV infection (10). Piglets receive maternal antibodies, mainly IgG and secretory IgA, from colostrum and milk of sows immunized with the PEDV vaccine, which provides piglets with passive immune protection against systemic infection and intestinal infection, respectively (10, 11). Although maternal antibodies prevent PEDV attaching to and entering target cells, they are ineffective against viruses inside host cells (12, 13). Therefore, new intracellular small molecules that specifically recognize and bind to PEDV must be generated to effectively prevent intracellular virus replication and control PED.

Single chain variable fragments (scFvs) are small molecule antibodies where the heavy chain variable region and the light chain variable region of the immunoglobulin molecule are linked by a short flexible peptide, commonly comprising glycine and serine residues (14, 15). When compared with monoclonal antibodies, scFvs not only retain intact monoclonal antibody specificity but also have low molecular weights, which provide stronger tumor penetration and higher molecular imaging sensitivity. Therefore, scFvs are ideal tools for tumor molecular imaging, disease therapy, and tracking targeted proteins *in vivo* (16–18).

PEDV is an enveloped, single-stranded positive-sense RNA virus, which belongs to the *Alphacoronavirus* genus in the *Coronaviridae* family (6). With an RNA genome of approximately 28 kb, the virus is composed of seven open reading frames (ORFs), a 5´ untranslated region (5′-UTR), and a 3´-UTR. The ORFs encode four structural proteins, including a spike (S) protein, an envelope (E) protein, a membrane (M) protein, and a nucleocapsid (N) protein, and three non-structural proteins, including replicase 1a, replicase 1b, and an accessory protein (19). Oral scFv administration to neonatal piglets targets the PEDV S protein and forms protection, which helps prevent and control PEDV infection (20). However, the S protein is frequently mutated, which challenges preventive and treatment strategies for PEDV (21, 22). As a structural protein, the N protein is the most abundant viral protein expressed in PEDV-infected cells and forms a ribonucleoprotein complex with the viral genomic RNA (23). During virion assembly, the N-RNA complex is incorporated into the virion by the interaction of N protein with M protein (24). It is highly conserved and plays important roles in PEDV replication (25–27). In addition, it involves regulate interferon (IFN) gene expression in host cells (28). Therefore, intracellular expressed scFvs targeting the PEDV N protein may exert effective inhibitory effects on viruses inside host cells.

Most scFvs bind only to extracellular pathogens, but not pathogens inside cells. Intracellular expressed scFvs that specifically bind to target proteins are important tools for studying protein molecular functions and preventing and treating disease. Adenovirus expression systems are widely used in gene therapy because they do not integrate foreign genes into host cell genomes, and they exhibit effective transduction abilities (29, 30). Adenovirus vectors are used in vaccine development to express antigens (31, 32) and in cancer therapy to express small molecules that target antigens such as scFvs and siRNAs (33–35). Recombinant adenoviruses can be administered intramuscularly, intravenously, intranasally or orally. Therefore, the adenovirus-mediated delivery of antigen-targeting scFvs into the body could provide protective effects and generate resistance to pathogen infection.

We previously reported that four scFvs, ZW1-16, ZW3-21, ZW1-41, and ZW4-16, specifically targeted the PEDV N protein, significantly suppressing PEDV replication, and upregulating IFN-λ1 expression in host cells (36). Herein, to investigate scFv effects on PEDV-induced diarrhea in piglets, four recombinant adenoviruses expressing scFvs against the PEDV N protein were generated. Recombinant adenovirus biosafety was assessed *in vivo*, and their distribution in piglet jejunums investigated. We also examined the protective effect of a combined scFv application in 3-day-old piglets with diarrhea caused by PEDV infection. Diarrhea severity in the oral recombinant adenovirus rAdV-scFv group was improved and sera IFN-λ expression upregulated when compared with control groups. Overall, adenovirus vector-mediated scFvs targeting of the PEDV N protein demonstrated effective preventive effects toward PEDV-induced diarrhea in piglets, therefore scFv-based therapeutic agents should be investigated to prevent and treat PED.

## Materials and methods

### Cell lines and virus

The HEK293 cell line kindly provided by Prof. Xuanming Yang (Shanghai Jiao Tong University, Shanghai, China) or African green monkey kidney cell line (Vero E6) was cultured in Dulbecco’s Modified Eagle’s Medium (DMEM; Gibco, Carlsbad, CA, USA) and 10% fetal bovine serum (FBS; Gibco). The IPEC-J2 was a gift from Prof. Jianxiong Xu (Shanghai Jiao Tong University, Shanghai, China) and cultured in DMEM/F12 and 10% FBS. Cell cultures contained 100 units/mL penicillin and 100 μg/mL streptomycin (Gibco). The PEDV (SH2012-5, GenBank accession number: MG837011.1) was propagated in Vero E6 cells.

### Preparation of recombinant adenovirus carrying anti-PEDV N scFvs

ScFvs targeting the PEDV N protein were previously generated and described (36). Briefly, a porcine recombinant scFv library was constructed and four scFvs targeting the PEDV N protein were generated. ScFvs were amplified using upstream primers containing the restriction enzyme *Sal*I (Thermo Fisher Scientific, Waltham, MA, USA) and downstream primers with *Hind*III (**Table 1**). PCR thermal cycling conditions were: 95°C for 5 min; 35 cycles of 95°C for 30 s, 62°C for 30 s, and 72°C for 55 s; and 72°C for 5 min. PCR products were purified and cloned into the shuttle vector pAdTrack-CMV (Addgene, Watertown, MA, USA). The linearized recombinant vector pAdTrack-CMV-scFv, digested with the restriction enzyme *Pme*I, was transformed into BJ5183 competent cells which contained the adenoviral backbone vector pAdEasy-1. Recombinant adenovirus plasmids in BJ5183 cells were selected using streptomycin and kanamycin, and termed pAdEasy-scFv (Addgene). After this, pAdEasy-scFvs were linearized with *Pac*I and transfected into HEK293 cells using TurboFect Transfection Reagent (Thermo Fisher Scientific). Until fluorescence and cytopathic effects appeared, cells were freeze-thawed three times to generate the recombinant adenovirus rAdV-scFv. A recombinant rAdV-wild-type adenovirus was used as a control.

**Table 1:** Primers used to amplify four scFvs for recombinant adenovirus vector construction.

Primer	Sequence (5’-3’)^a^
TrackZW1-16-B*Sal*I	TCGATGTCGACATGTACCCATACGATGTTCCAGATTACGCTGAGGAGAAGCTGGTGGAGTCCGG
TrackZW1-16-F*Hind*III	TAACTAAGCTTTCAGAGCTCGTCCTTTTTGAGTTCCAGCTTGGTCCC
TrackZW3-21-B*Sal*I	TCGATGTCGACATGTACCCATACGATGTTCCAGATTACGCTGAGGTGAAGCTGGTGGAGTCTGG
TrackZW3-21-F*Hind*III	TAACTAAGCTTTCAGAGCTCGTCCTTTTTGATCTCCAGCTTGGTCCC
TrackZW1-41-B*Sal*I	TCGATGTCGACATGTACCCATACGATGTTCCAGATTACGCTGAGGAGAAGCTGGTGGAGTCTGG
TrackZW1-41-F*Hind*III	TAACTAAGCTTTCAGAGCTCGTCCTTTTTGAGCTCCAGCTTGGTCCC
TrackZW4-16-B*Sal*I	TCGATGTCGACATGTACCCATACGATGTTCCAGATTACGCTGAGGTGAAGCTGGTGGAGTCTGG
TrackZW4-16-F*Hind*III	TAACTAAGCTTTCAGAGCTCGTCCTTGAGGACGGTCAGATGGGTCC

a SalI and HindIII restriction sites are underlined.

### scFv expression in HEK293 or IPEC-J2 cells

HEK293 or IPEC-J2 cells were seeded in 6-well plates and cultured at 37°C in 5% CO_2_ until 80% confluence. Cells were then infected with the recombinant adenovirus rAdV-scFv or rAdV-wild-type at a multiplicity of infection of 1 and 100, respectively. At 36 h post-infection, cells were lysed in RIPA lysis buffer (Beyotime Biotechnology, Shanghai, China) and scFv expression analyzed by western blotting. Total proteins were separated using sodium dodecyl sulfate-polyacrylamide gel electrophoresis and transferred to nitrocellulose membranes (Santa Cruz Biotechnology, Santa Cruz, CA, USA). After blocking in 5% skim milk, membranes were incubated with a primary mouse monoclonal anti-HA antibody (CMCTAG, Milwaukee, WI, USA) overnight at 4°C, and then incubated with a horseradish peroxidase (HRP)-conjugated Affinipure goat anti-mouse IgG (H + L) antibody (Jackson ImmunoResearch, West Grove, PA, USA) at 37°C for 1 h. Protein bands were revealed using Immobilon Western Chemiluminescent HRP Substrate (Merck-Novagen, Darmstadt, Germany).

### scFv expression in the jejunum

Twenty-seven 3-day-old piglets without the PEDV vaccine were purchased from a commercial non-PEDV epidemic pig farm (Wujiang Tianyu Biological Technology Co., Ltd, Suzhou, China). Piglets were randomly divided into nine piglets/groups. Group 1 piglets were orally given 2 mL 10^10^TCID_50_ (median tissue culture infective dose) recombinant adenovirus rAdV-ZW (equal quantities of rAdV-ZW1-16, rAdV-ZW3-21, rAdV-ZW1-41, and rAdV-ZW4-16). Piglets in groups 2 and 3 were orally given 2 mL 10^10^TCID_50_ recombinant adenovirus rAdV-wild-type and 2 mL PBS, respectively. Three piglets from each group were humanely sacrificed on days 2, 14, and 18 after oral administration, respectively. A jejunum section was excised and lysed in RIPA lysis buffer to evaluate scFv expression by western blotting. Additionally, a jejunum section was fixed in paraformaldehyde and paraffin-embedded tissue sections were prepared to analyze scFv expression using indirect immunofluorescence and immunohistochemistry assays (see below).

### Assessing recombinant adenovirus biosafety

Nine 3-day-old non-PEDV infected piglets were purchased, randomly divided into three groups, and treated as described. Before oral recombinant adenovirus administration, piglet weights were recorded. Rectum temperatures were also measured and recorded every day after oral administration. Feces were collected at 1 day intervals to measure scFv levels using indirect enzyme-linked immunosorbent assay (ELISA). The following method (37) was used: 1 mL PBS buffer containing 20 mM phenylmethylsulfonyl fluoride (PMSF, Beyotime Biotechnology) was added to 0.5 g feces. After intense vortexing, the mixture was shaken at 200 × *g* for 1 h at 4°C and centrifuged at 8,000 × *g* for 10 min to collect the supernatant which was filtered through a 0.45 μm filter and used to coat a 96-well plate. The plate was incubated overnight at 4°C and then blocked in 4% bovine serum albumin (BSA) at 37°C for 2 h. A mouse monoclonal anti-HA antibody was added to each well and incubated at 37°C for 2 h. A secondary HRP-conjugated Affinipure goat anti-mouse IgG (H + L) antibody was added to each well and incubated at 37°C for 1 h. After terminating the reaction, the optical density at 450 nm was measured using a Multiskan™ FC Microplate Photometer (Thermo Fisher Scientific). All piglets were weighed on day 10 after oral administration and percentage weight gains calculated.

Piglets were monitored for 10 days and then humanely sacrificed. Jejunums were collected and scFv expression analyzed by western blotting and indirect immunofluorescence and immunohistochemistry assays. Hearts, livers, spleens, lungs, kidneys, stomachs, small intestines, and large intestines were excised and fixed in paraformaldehyde. Pathological sections were generated and tissue morphology observed using hematoxylin-eosin (H&E) staining. IFN-γ and interleukin-4 (IL-4) quantification in jejunum samples was performed as described below.

### Cytokine analysis

Jejunums from piglets orally treated with recombinant adenovirus were collected and frozen in liquid nitrogen on day 10 and then ground in physiological saline solution. After centrifugation at 1,000 × *g* for 10 min, the supernatant was collected and IFN-γ and IL-4 levels measured using commercial ELISA kits according to manufacturer’s recommendations (Shanghai Enzyme-linked Biotechnology Co., Ltd., Shanghai, China).

Sera were collected on day 3 after PEDV challenge and IL-6, IL-8, IL-12, TNF-α, and IFN-λ levels quantified using commercial ELISA kits (Shanghai Enzyme-linked Biotechnology Co., Ltd.).

### Immunofluorescence assay

An indirect immunofluorescence assay was performed to evaluate scFv infection efficiency and distribution in jejunum samples. Jejunal sections were dewaxed, rehydrated, and subjected to antigen retrieval. After blocking in 3% BSA at room temperature for 30 min, sections were incubated with a primary mouse monoclonal anti-HA antibody overnight at 4°C, followed by incubation with a Cy3-conbjugated Affinipure goat anti-mouse IgG (H + L) secondary antibody at room temperature for 1 h. After cell nuclei were stained with 4´,6-diamidino-2-phenylindole (DAPI), sections were analyzed using a Nikon Eclipse microscope (Nikon, Tokyo, Japan). PEDV N protein expression was detected by staining sections with a rabbit polyclonal anti-PEDV N protein antibody and then a HRP-conjugated Affinipure goat anti-rabbit IgG (H + L) secondary antibody. Sections were observed under a Nikon Eclipse microscope (Nikon) following incubation with fluorescein isothiocyanate (FITC)-tyramide signal amplification.

### Immunohistochemistry assay

After dewaxing, rehydrating, and antigen retrieval, endogenous peroxidases were blocked in 3% hydrogen peroxide for 25 min. Jejunal sections were blocked in 3% BSA and probed with a primary mouse monoclonal anti-HA antibody overnight at 4°C. After incubation with a Cy3-conjugated Affinipure goat anti-mouse IgG (H + L) secondary antibody, samples were assessed using a Dako REAL EnVision Detection System (Dako, Glostrup, Denmark) at room temperature for 5 min. Sections were washed in ddH_2_O to terminate reactions and stained in Harris hematoxylin for 3 min. Then, samples were dehydrated, cleared, mounted, and observed and imaged under a microscope (Nikon).

### Responses of PEDV-infected piglets orally administrated rAdV-scFv

As shown in **Table 2**, 15 × 3-day-old piglets were randomly divided into five groups with three piglets/group. Group I and IV piglets were orally treated with 2 mL 10^10^TCID_50_ rAdV-ZW. Group II piglets orally received 2 mL 10^10^TCID_50_ rAdV-wild-type. Group III and V piglets were treated orally with 2 mL PBS (controls). All piglets were retreated at 2 day intervals. At 6 h after the second oral recombinant adenovirus administration, groups I–III piglets were orally challenged with 1 mL 5×10^5^TCID_50_ PEDV, while groups IV and V piglets were orally challenged with 1 mL PBS. Piglets were monitored for 7 days, and clinical signs and symptoms, including body weight, body temperature, and diarrhea, were evaluated. Average weight gain and rectum temperatures were recorded every day during studies. Fecal rectal samples were used to assess diarrhea severity. Fecal consistency was evaluated using a scoring scale of 0–3: 0 = solid feces, normal; 1 = pasty feces, slight diarrhea; 2, semi-liquid feces, moderate diarrhea; and 3, liquid feces, severe diarrhea (38, 39).

**Table 2:** PEDV challenge in piglets: experimental design.

Group	Number of piglets	Recombinant adenovirus category	Dose	PEDV category	Dose	Route of administration
I	3	rAdV-ZW	2 mL (10^10^TCID_50_)	PEDV	1 mL (5×10^5^TCID_50_)	Oral
II	3	rAdV-wild	2 mL (10^10^TCID_50_)	PEDV	1 mL (5×10^5^TCID_50_)	Oral
III	3	PBS	2 mL	PEDV	1 mL (5×10^5^TCID_50_)	Oral
IV	3	rAdV-ZW	2 mL (10^10^TCID_50_)	PBS	1 mL	Oral
V	3	PBS	2 mL	PBS	1 mL	Oral

### Determining viral RNA in feces

Fecal samples were collected every day after the PEDV challenge. Viral RNA was extracted using the E.Z.N.A. Stool RNA Kit (Omega Bio-tek, Norcross, GA, USA) and served as a template for complementary DNA (cDNA) synthesis using the PrimeScript™ RT reagent Kit with gDNA Eraser (Perfect Real Time) (Takara Bio, Shiga, Japan). PEDV M gene expression was analyzed by reverse transcription (RT)-quantitative PCR (qPCR) to quantify virus production in feces (36).

### Statistical analysis

Data were presented as the mean ± standard error of the mean (SEM). Statistical analyses were performed using Origin 8.0 software (OriginLab, Northampton, USA) and GraphPad Prism v8.0 software (GraphPad Software Inc., San Diego, CA, USA). P < 0.05 was considered statistically significant.

## Results

### Preparation of recombinant adenovirus rAdV-scFvs

**Figure 1:**
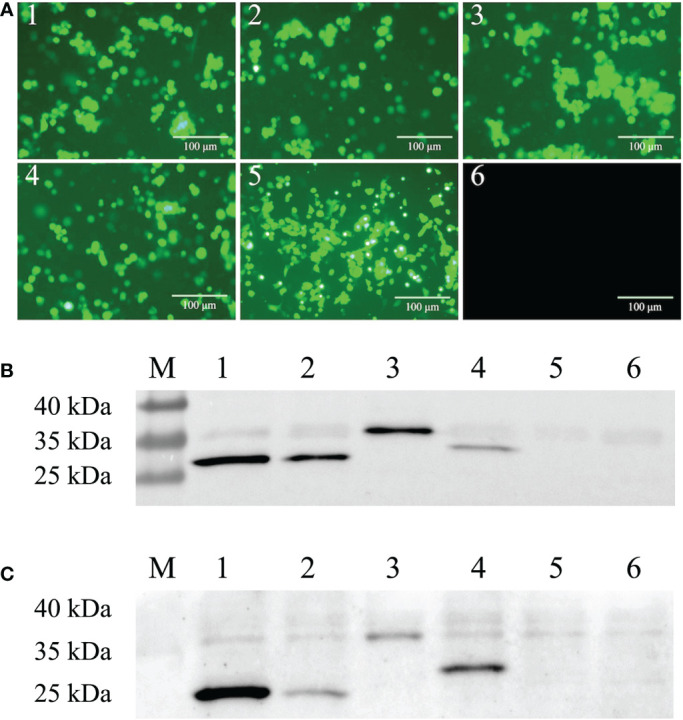
Construction of recombinant adenovirus rAdV-scFvs and expression in cells. **(A)** Fluorescent images of rAdV-scFv-infected HEK293 cells, rAdV-wild-type infected HEK293 cells, and uninfected HEK293 cells. HEK293 cells were infected with rAdV-ZW1-16, rAdV-ZW3-21, rAdV-ZW1-41, rAdV-ZW4-16, and rAdV-wild-type (1–5) and observed under fluorescence microscopy; uninfected HEK293 cells were used as MOCK controls (6). Scale bar = 100 μm. **(B, C)** Western blots showing scFv expression in HEK293 **(B)** and IPEC-J2 cells **(C)** infected with rAdV-scFvs and rAdV-wild type, respectively. Lane M, protein molecular weight marker; lanes 1–4, whole cell lysates from infected cells expressing rAdV-ZW1-16, rAdV-ZW3-21, rAdV-ZW1-41, and rAdV-ZW4-16, respectively; lane 5, whole cell lysate from infected cells plus rAdV-wild-type; lane 6, uninfected cell lysate.

Four scFvs, ZW1-16, ZW3-21, ZW1-41, and ZW4-16, targeting the PEDV nucleocapsid (N) protein were generated from a porcine recombinant scFv library (36). Genes of the scFvs were amplified, cloned into a replication-defective Ad5 vector, and packaged into HEK293 cells (**Supplementary Figure 1**). A green fluorescent protein (GFP) gene incorporated into the adenoviral backbone allows direct observation of the efficiency of viral infection (40). Green fluorescence was observed in cells infected with rAdV-ZW1-16, rAdV-ZW3-21, rAdV-ZW1-41, rAdV-ZW4-16, and rAdV-wild-type (**Figure 1A**). Next, the expression of four scFvs was analyzed by western blotting. The prominent bands of ZW1-16, ZW3-21, and ZW4-16 were approximately 30 kDa, and that of ZW1-41 was approximately 35 kDa (**Figure 1B**). The sizes of scFvs were consistent with our previous data (36). To measure scFv expression in porcine cells, IPEC-J2 cells were infected with rAdV-scFvs; four major bands at approximately 30-35 kDa were detected (**Figure 1C**). Thus, four rAdV-scFvs encoding scFv genes were successfully constructed and expressed in IPEC-J2 cells.

### The rAdV-scFvs biosafety

**Figure 2:**
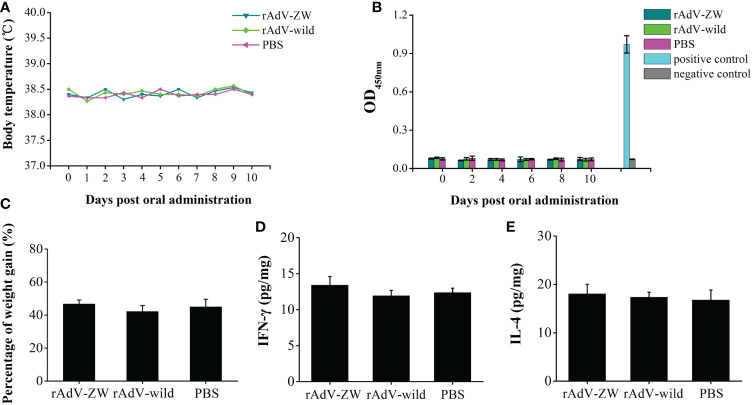
Biosafety rAdV-ZW testing. Three-day-old piglets were orally administrated 2 mL 10^10^TCID_50_ rAdV-ZW, 2 mL 10^10^TCID_50_ rAdV-wild-type, and 2 mL PBS. **(A)** The rAdV-ZW effects on rectal temperature. After administration, piglet temperatures were measured daily. **(B)** Viral shedding determined by ELISA. After administration, feces samples were collected at one day intervals. **(C)** Piglet percentage weight gain after rAdV-ZW administration. Jejunal **(D)** IFN-γ and **(E)** IL-4 expression by ELISA at day 10 after oral administration. Values are presented as the mean ± standard error of the mean (SEM).

**Figure 3:**
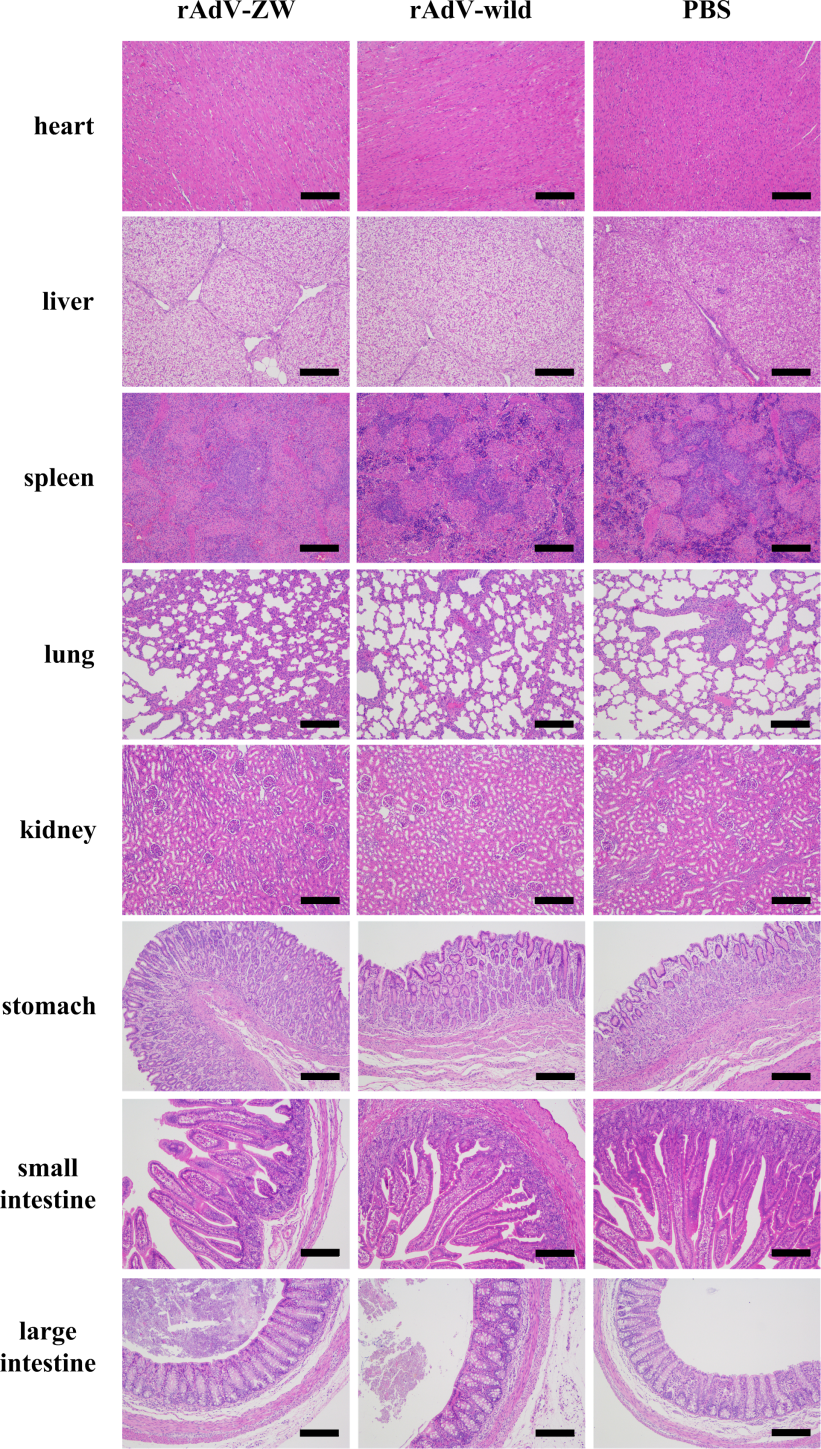
Histopathological analyses of different tissues after orally administrating rAdV-ZW, rAdV-wild-type, or PBS to piglets. Hematoxylin & eosin (H&E) staining of heart, liver, spleen, lung, kidney, stomach, small intestine, and large intestine. Scale bar = 200 μm.

To explore the biosafety of recombinant adenovirus mediated the scFvs *in vivo*, 3-day-old piglets were orally administered rAdV-ZW (equal quantities of rAdV-ZW1-16, rAdV-ZW3-21, rAdV-ZW1-41, and rAdV-ZW4-16), rAdV-wild-type, or PBS. During studies, no adverse clinical effects were observed. Rectal temperatures were measured every day and were always approximately 38.5°C (**Figure 2A**). No significant differences in body temperatures among groups were identified. Piglets were weighed and weight gain rates during studies were calculated. The average percentage weight gain was approximately 40%, with no statistically significant differences (**Figure 2C**). Fecal samples were collected every day to measure scFv levels using indirect ELISA. Fecal samples from all groups were negative for scFv levels, indicating that adenovirus-mediated scFvs were not environmentally released *via* defecation (**Figure 2B**). To assess if rAdV-ZW induced Th1 and Th2 immune responses, IFN-γ and IL-4 expression levels in jejunum samples were analyzed by ELISA. No significant differences in IFN-γ and IL-4 expression were identified among groups (**Figures 2D, E**). Additionally, histological morphology analyses of heart, liver, spleen, lung, kidney, stomach, small intestine, and large intestine revealed no abnormalities (**Figure 3**). Collectively, adenovirus-mediated anti-PEDV N scFv expression in the jejunum was biosafe for piglets.

### Jejunal expression of adenovirus-mediated scFvs

**Figure 4:**
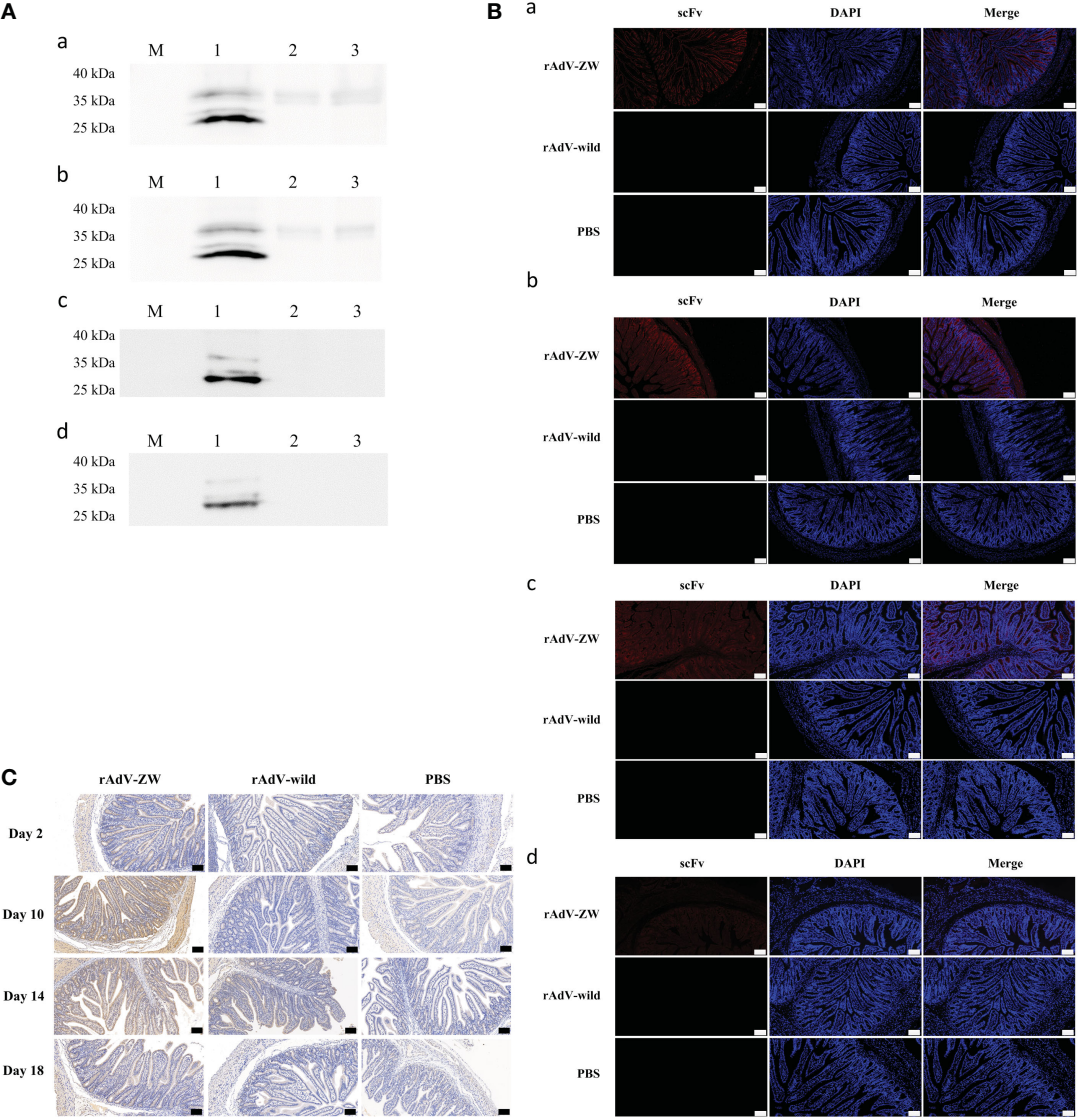
Jejunal expression of four scFvs. After rAdV-ZW, rAdV-wild-type or PBS oral administration, jejunums were collected on days 2 (**Aa**, **Ba**), 10 (**Ab**, **Bb**), 14 (**Ac**, **Bc**), and 18 (**Ad**, **Bd**). **(A)** Western blotting of scFv expression in jejunum tissue showing several bands at approximately 30-35 kDa. Lanes 1–3, jejunum tissue lysate from piglets administrated rAdV-ZW (lane 1), rAdV-wild-type (lane 2), and PBS (lane 3). **(B)** Immunofluorescence analysis showing scFv expression in jejunum tissue. Jejunum paraffin sections were incubated with a primary mouse monoclonal anti-HA antibody to detect HA-tagged scFvs, and then stained with DAPI to observe nuclei. Red fluorescence represents scFvs and blue represents nuclei. Scale bar = 100 μm. **(C)** Immunohistochemical jejunum tissue staining to analyze scFv expression. Brown staining indicates scFvs and blue represents hematoxylin-stained nuclei. Scale bar = 100 μm.

Previous reports revealed that scFv cocktails exhibited better therapeutic effect than single scFvs (41, 42). In our study, a four-scFv cocktail of ZW1-16, ZW3-21, ZW1-41, and ZW4-16 was used. To analyze scFv expression in the jejunum, piglets were orally administered recombinant adenovirus rAdV-ZW or rAdV-wild-type. On days 2, 10, 14, and 18 after oral administration, jejunums were collected and analyzed by western blotting, immunofluorescence, and immunohistochemistry. As shown in **Figure 4A**, scFvs were successfully expressed in the jejunum of the rAdV-ZW group, with protein sizes consistent with *in vitro* data (**Figures 1B, C**). No specific protein bands were identified in orally administered rAdV-wild-type and PBS piglets. Immunofluorescence analysis showed that scFv fluorescence signals were distributed in epithelial cells mainly on jejunum villi surfaces at day 2 after rAdV-ZW oral administration (**Figure 4Ba**). Notably, scFvs were expressed in cells of the entire jejunum mucosa, including epithelium, underlying lamina propria, muscularis mucosa, and submucosa, at day 10 after oral administration (**Figure 4Bb**). Over time, the signal of scFv expression gradually weakened (**Figures 4Bc, Bd**). The expression of scFvs in jejunum could still be detected at day 18 after oral administration (**Figure 4Bd**). Additionally, immunohistochemical analyses was used to determine scFv expression and distribution in the jejunum of piglets mediate by recombinant adenovirus. ScFv expression on day 10 were higher than on days 2, 14, and 18 in the rAdV-ZW oral administration group (**Figure 4C**). These immunohistochemical data were consistent with immunofluorescence data. No fluorescence signals were observed in jejunum tissue from rAdV-wild-type and PBS groups. Collectively, western blotting, immunofluorescence, and immunohistochemistry data confirmed that scFvs were successfully expressed in piglet jejunums.

### *In vivo* scFv expression alleviates clinical symptoms in PEDV-infected piglets

**Figure 5:**
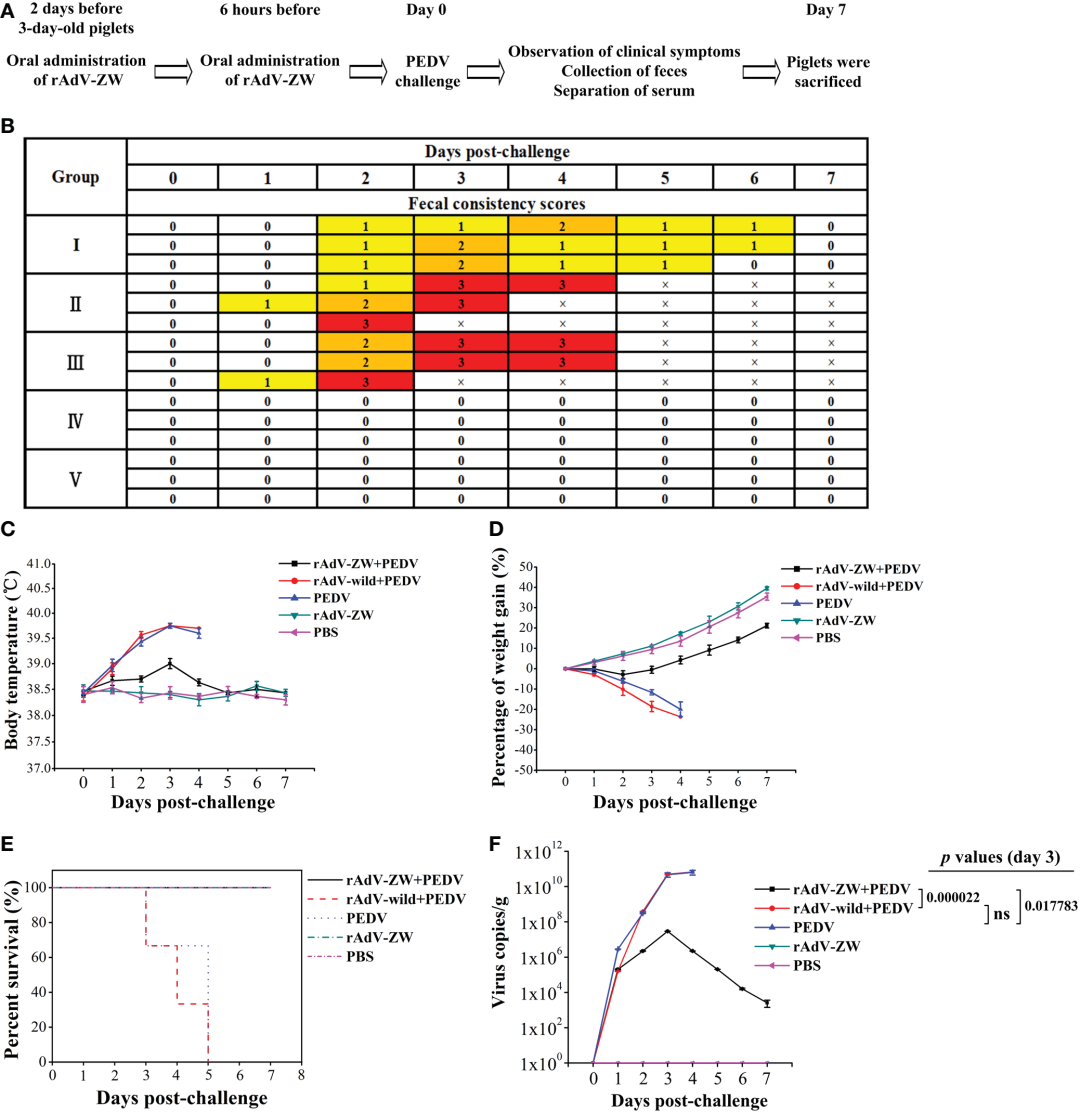
Evaluating the protective effects of scFvs on PEDV-induced diarrhea in piglets. **(A)** Experimental design showing the oral administration of rAdV-ZW, PEDV challenge, and how we evaluated protective effects in piglets. **(B)** After PEDV challenge, fecal consistency scores were measured to evaluate the effects of rAdV-ZW on diarrhea severity. Fecal consistency was evaluated using a 0-3 scoring scale: 0 = solid feces; 1 = pasty feces; 2 = semi-liquid feces; and 3 = liquid feces. **(C)** Effects of rAdV-ZW on rectal temperatures of piglets challenged with PEDV. Rectal temperatures, induced by PEDV in piglets *via* the oral administration of rAdV-wild-type or PBS, were significantly increased; the highest temperature was approximately 40°C, and rAdV-ZW administration delayed and attenuated increased rectal temperatures induced by PEDV in piglets. **(D)** Piglet weight gain percentages during studies. Piglets were weighed before PEDV challenge and at 7 dpc, with weight gain percentages shown. PEDV-infected piglets upon oral rAdV-wild-type or PBS administration lost approximately 20% of their body weight. **(E)** Piglets were observed for 7 days after challenge to assess the effects of rAdV-ZW on survival rates. After PEDV infection, piglets orally administered rAdV-wild-type or PBS died, while those orally administered rAdV-ZW survived. **(F)** PEDV feces quantification; fecal samples were collected daily and viral RNA extracted to analyze PEDV production by RT-qPCR using specific PEDV M gene primers. Values were presented as the mean ± standard error of the mean (SEM). Ns = not significant (P > 0.05).

To explore scFv protective effects, piglets were orally administered rAdV-ZW and challenged with the prevalent PEDV strain following the indicated scheme (**Table 2** and **Figure 5A**). Clinical symptoms were observed and recorded. Group II and III piglets had slight diarrhea at 1-day post-challenge (dpc). Over time, diarrhea symptoms became more significant. All group II and III piglets developed severe diarrhea at 2 or 3 dpc (**Figure 5B**). As shown in **Figure 5B**, group I piglets had slight diarrhea at 2 dpc and moderate diarrhea at 3 or 4 dpc, but no severe diarrhea occurred and piglets had no diarrhea at 7 dpc. Body temperatures in group II and III piglets increased gradually and almost reached 40°C on day 3 after challenge (**Figure 5C**). Body temperatures in group I piglets increased slightly after challenge, but then gradually returned to normal (**Figure 5C**). Thus, the degree of PEDV-induced diarrhea of piglets prevented with rAdV-ZW in group I was reduced.

Piglets in control groups IV and V had a normal mental state, normal appetites, and gained 35%–40% of their body weight (**Figure 5D**). After PEDV challenge, group II and III piglets were depressed and their appetite decreased. By the time of death, they had lost approximately 20% of their body weight. The mental state of group I piglets was hardly affected; body weights were almost unchanged in the first 3 days after challenge; they gradually increased from the fourth day and increased by approximately 20% at 7 dpc (**Figure 5D**). Statistical survival rate analyses showed that all piglets in groups II and III died at 3–5 dpc, whereas survival rate in the other three groups was 100% (**Figure 5E**). Therefore, scFvs targeting the PEDV N protein alleviated diarrhea in piglets, which was caused by PEDV infection.

### scFvs mediate virus shedding reductions in PEDV-infected piglets

Fecal samples were collected every day (**Table 2** and **Figure 5A**) to determine viral shedding by RT-qPCR. As shown in **Figure 5F**, PEDV virions were detected in feces from groups I–III on the first day after challenge, while viral RNA levels in groups II and III increased sharply, reaching 10^10^ copies/g at 3 and 4 dpc. Viral RNA levels in group I increased gradually and peaked at 10^7^ copies/g at 3 dpc, but these levels were significantly lower in groups II and III (P < 0.05). Over time, viral RNA levels in group I gradually decreased to 10^3^ copies/g at 7 dpc. No PEDV virions were detected in feces from groups IV and V (**Figure 5F**). These results showed that scFvs targeting the PEDV N protein significantly inhibited virus replication in PEDV-infected piglets.

### scFvs expressed in the jejunum co-localize with the PEDV N protein

**Figure 6:**
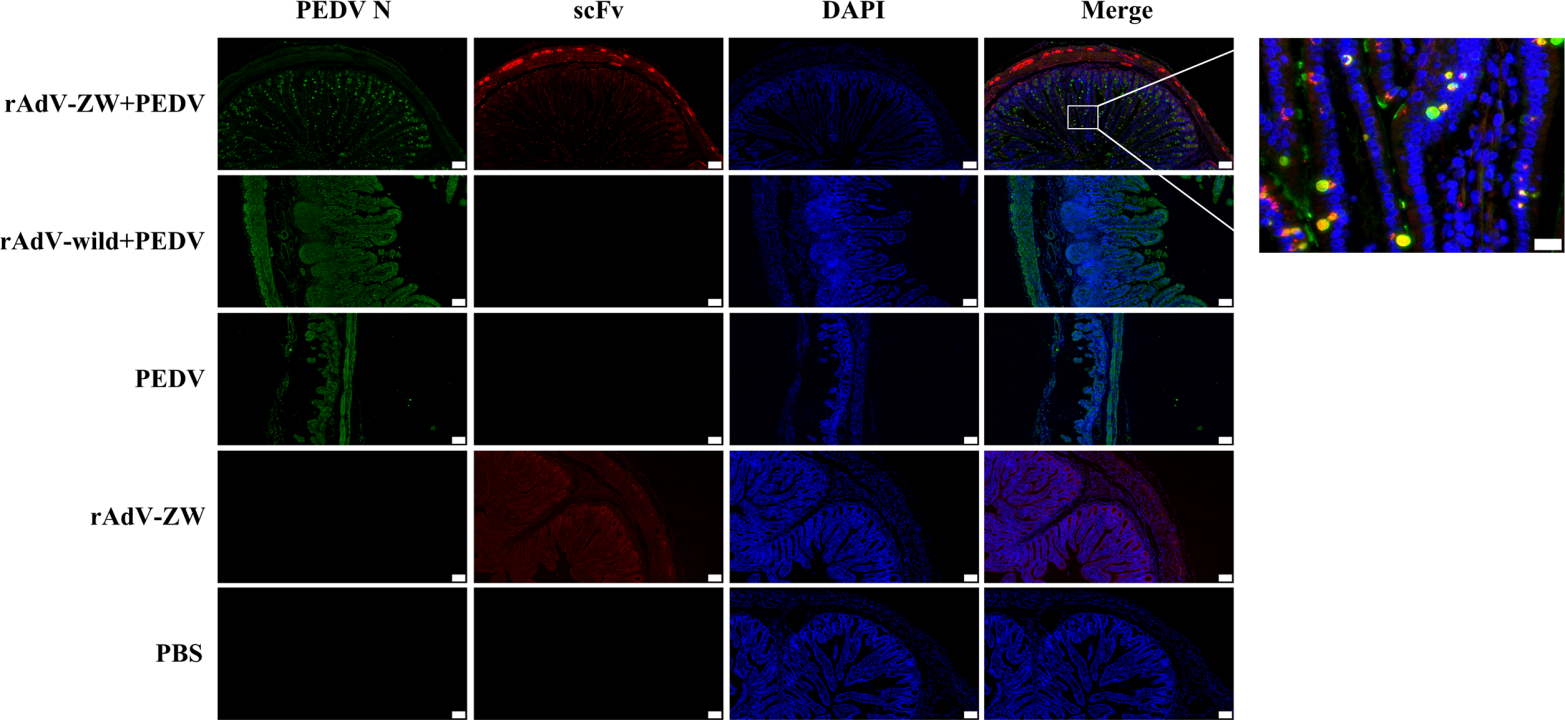
ScFv and PEDV N protein expression in jejunum by immunofluorescence. Jejunum samples were collected from piglets orally administrated rAdV-ZW after challenge with PEDV. Paraffin sections were stained with FITC, CY3, and DAPI and observed at 100× magnification (left). Scale bar = 100 μm. (Right) (630×) Enlarged framed area in (left). Scale bar = 20 μm. Green represents the PEDV N protein; red represents scFvs, and blue indicates DAPI-stained nuclei. In merged images, orange indicates ZW co-localization (including ZW1-16, ZW3-21, ZW1-41, and ZW4-16) with the PEDV N protein.

In our previous study, we reported that the four scFvs co-localized with the wild-type N protein in PEDV-infected cells (36). To determine scFv and wild-type N protein expression and distribution *in vivo*, jejunums from piglets orally administered rAdV-ZW and challenged with the prevalent PEDV strain (**Table 2** and **Figure 5A**) were collected, fixed, and paraffinized. Both scFv and PEDV N protein expression were analyzed using indirect immunofluorescence assays (**Figure 6**); scFvs were highly expressed in the jejunum in groups I and IV, while no fluorescence (red) was observed in groups II, III, and V. Interestingly, PEDV was evenly distributed in the whole small intestine in group II and III piglets. However, when piglets were orally administrated rAdV-ZW in advance and then challenged with PEDV, PEDV was mainly distributed on jejunum villi surfaces, and scFvs were distinctly co-localized with PEDV N proteins in the jejunum. Together, these data suggested that PEDV infected epithelial cells on jejunum villi surfaces after viral challenge, and scFvs expressed in jejunum epithelial cells specifically bound PEDV N proteins, resulting in inhibited PEDV replication and decreased intact PEDV virions, with PEDV unable to infect more cells.

### scFvs ameliorate intestinal pathological damage caused by PEDV infection in piglets

**Figure 7:**
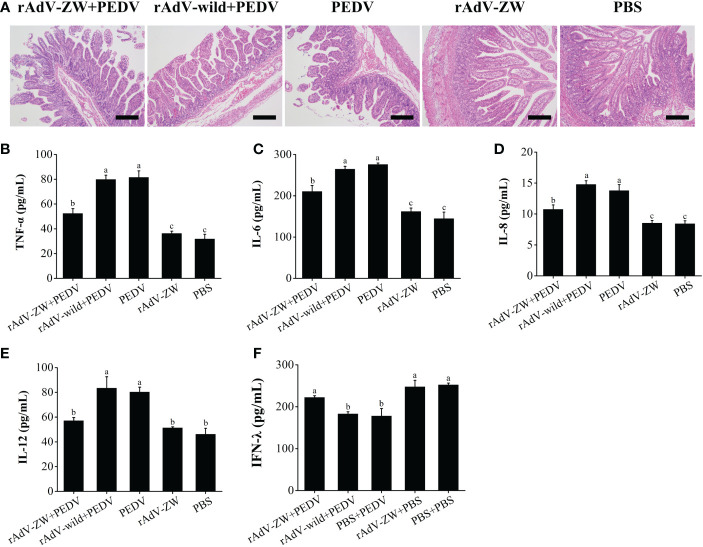
Histomorphological observations in jejunum and cytokine production levels in piglets. **(A)** Jejunum tissue underwent H&E staining to assess the effects of rAdV-ZW on jejunum pathological changes in piglets infected with PEDV. H&E staining showed that rAdV-ZW reduced jejunal damage severity. Scale bar = 200 μm. **(B–E)** Pro-inflammatory sera cytokine expression in piglets. At 3 dpc, TNF-α, IL-6, IL-8 and IL-12 serum levels were assayed using indirect ELISA. **(B)** TNF-α expression, **(C)** IL-6 expression, **(D)** IL-8 expression, **(E)** IL-12 expression, and **(F)** IFN-λ expression profiles in piglet sera at 3 dpc. Values were presented as the mean ± standard error of the mean (SEM). Values indicated by different letters are significantly different (P < 0.05). No significant differences between values are indicated by identical letters.

Histological analyses showed that jejunum villi were considerably atrophied, with thinning intestinal walls, mesenteric hyperemia, necrosis, and intestinal villus epithelial cell shedding, with intestinal mucosa structures severely damaged in group II and III piglets (**Figure 7A**). The jejunum villi were mildly atrophied, and the intestinal mucosa structure was slightly damaged of piglets prevented by the scFvs against the PEDV N protein before infection with PEDV in group I. Jejunum morphology in control groups IV and V was normal. These pathological observations showed that jejunum damage caused by PEDV infection was significantly alleviated when scFvs directed against the PEDV N protein were expressed in the jejunum.

### scFvs-mediate pro-inflammatory cytokine regulation and IFN-λ1 expression

Serum pro-inflammatory cytokine levels, including TNF-α, IL-6, IL-8, and IL-12 were evaluated by ELISA (**Figures 7B–E**). Serum TNF-α, IL-6, IL-8, and IL-12 levels in group II and III piglets infected were significantly increased when compared with control groups IV and V (P < 0.05). TNF-α, IL-6, and IL-8 serum expression levels in PEDV-infected group I piglets treated with rAdV-ZW were significantly lower than in groups II and III (P < 0.05), although levels were significantly higher than in control groups IV and V (P < 0.05). Serum IL-12 levels in PEDV-infected group I piglets treated with rAdV-ZW were significantly decreased when compared with groups II and III (P < 0.05), with no significant differences between groups I, IV, and V (P > 0.05).

We previous showed that IFN-λ1 expression levels were significantly up-regulated after the PEDV N protein was blocked by the four scFvs in PEDV-infected IPEC-J2 cells (36). To examine if IFN-λ expression level in piglets infected with PEDV changed after scFvs were delivered to the jejunum, serum was collected and processed using indirect ELISA. As shown in **Figure 7F**, IFN-λ serum expression in PEDV-infected group II and III piglets was significantly down-regulated when compared with control groups IV and V (P < 0.05), whereas expression was up-regulated in PEDV-infected group I piglets treated with rAdV-ZW (P < 0.05). Thus, the four scFvs eliminated the antagonistic effects of PEDV infection toward IFN-λ expression by blocking PEDV N protein expression *in vivo*.

## Discussion

In recent years, vaccination has become a most effective approach controlling PEDV; traditional strategies have mainly included PEDV inactivated vaccines, live attenuated vaccines, and genetically engineered vaccines (43, 44). However, because inactivated vaccines cannot be replicated, pigs must be immunized multiple times with large doses. Also, attenuated live vaccines may revert to wild-type PEDV strains, and most genetically engineered vaccines currently developed use the PEDV S protein as antigen, however, the mutational probability of the S gene in variant strains is very high, resulting in the prevention and control effect of PED caused by PEDV variant strain is not ideal (8, 22, 45). Therefore, novel effective strategies are required against PEDV.

Some scFvs were previously evaluated for their efficacy in detecting, preventing, and treating disease (18, 46, 47). The scFvs targeting the PEDV S protein were effective in controlling piglet diarrhea (20). The PEDV N protein is a structural protein and highly conserved when compared with the S protein. It has key functions in the viral life cycle and is involved in interactions between the virus and host cells. Coronavirus N protein contributes to viral RNA synthesis by interacting with nonstructural protein 3 (nsp3), a component of the replication-transcription complex (48). It was shown that the PEDV N protein promoted viral replication (25) and different regulatory factors in host cells could suppress viral replication by degrading the N protein (49, 50). Because the protein has multiple functions, specific protein targeting by scFvs is an ideal way to inhibit viral infection. We constructed a recombinant scFv library from the spleens of pigs immunized with purified recombinant PEDV N protein, and identified four scFvs, ZW1-16, ZW3-21, ZW1-41, and ZW4-16, which specifically bound to the PEDV N protein (36). ScFvs co-localized with the wild-type PEDV N protein, significantly suppressed PEDV replication, and observably upregulated IFN-λ1 expression in PEDV-infected cells (36).

Adenovirus is a double-stranded DNA virus with an icosahedral capsid and no envelope. Adenovirus vectors are effective for *in vivo* gene delivery systems (51, 52); human adenovirus type 5 is the most commonly used vector for vaccine preparations and gene therapy (40, 53). In our study, the adenoviral plasmid pAdEasy-1 was replication defective and contained most of the human adenovirus type 5 genome, except for E1 and E3 genes, and was used as the backbone to generate the recombinant adenovirus rAdV-scFv. Four recombinant adenoviruses were successfully packaged into HEK293 cells and scFvs successfully expressed in rAdV-scFv-infected IPEC-J2 cells.

Previous studies have shown that a cocktail of several scFvs targeting different molecular epitopes could enhance sensitivity of scFv-based disease detection and improve disease prevention potency when compared with individual scFvs (54, 55). Therefore, we combined four scFvs against the PEDV N protein to explore their efficacy in preventing PEDV infection. We evaluated rAdV-scFvs biosafety by measuring piglet body temperatures, body weights, scFv levels in feces, IFN-γ and IL-4 expression in the jejunum, and pathological changes in eight tissues after oral administration. No significant differences were observed in body temperatures, weight gain rates, and IFN-γ and IL-4 expression levels in jejunum when compared with control piglets. No scFvs were detected in feces and no lesions were identified in tissue sections of rAdV-ZW-treated piglets.

Recombinant adenoviruses deliver genes into the body in several ways. Orally administered adenovirus vaccines may induce mucosal immune responses in animals (56, 57). Because PEDV mainly infects porcine small-intestinal epithelial cells, in our study, rAdV-scFvs directly delivered four scFvs to piglet intestinal tissues *via* oral administration. We investigated scFv distribution in the jejunum using western blotting, immunofluorescence, and immunohistochemical analysis at days 2 and 10 after oral administration - expression of the four scFvs was detected at both time points. Immunohistofluorescence and immunohistochemical analyses showed scFvs were mainly expressed in cells on jejunum villi surfaces on day 2 and distributed to the whole jejunum by day 10.

After confirming scFvs were expressed in porcine jejunum tissue and biosafe for piglets, we evaluated the prophylactic effects of rAdV-ZW on diarrhea caused by PEDV. Immunofluorescence analyses showed that scFvs were co-localized with the PEDV N protein in jejunum epithelial cells in piglets challenged with PEDV, consistent with vero E6 cells in a previous study (36). Surprisingly, after challenge, PEDV was almost uniformly distributed in all jejunum cells in piglets administered rAdV-wild or PBS, whereas, PEDV was mainly detected in jejunum villi cells surfaces in piglets administered rAdV-ZW. We speculated that scFvs expressed in epithelial cells bound to the N protein of intracellular PEDV, inhibiting PEDV replication, not forming complete virions in assembly processes, and not generating enough viruses to infect other cells. Consequently, epithelial cells infected by PEDV were mainly present on villi surfaces.

After PEDV challenge, piglets administrated rAdV-wild or PBS showed elevated body temperatures and severe diarrhea, and became thinner during studies, while piglets treated with rAdV-ZW showed reduced clinical symptoms with a slight increase in body temperature, moderate diarrhea, and a body weight gain of approximately 20%. Thus, scFvs already expressed in intestinal epithelial cells provided effective protection against viral infection. PEDV infection in piglets treated with rAdV-wild or PBS induced the severe destruction of jejunal villi and 100% mortality in both groups. In contrast, jejunal villi in piglets orally administrated rAdV-ZW were slightly damaged, and all piglets survived. Therefore, a four-scFv cocktail prevented PEDV infection in piglets.

The PEDV N protein promotes viral replication and accelerates higher titer viral growth (25, 26). Several studies reported that PEDV replication was suppressed when the N protein was degraded (49, 58, 59). Our scFvs targeting the PEDV N protein significantly suppressed PEDV replication in PEDV-infected cells, as described in our previous study (36). Previous studies indicated that PEDV infection in piglets caused fecal viral RNA shedding (39, 60). In our study, fecal samples were analyzed to assess PEDV RNA shedding titers; no PEDV was detected in feces from control animals administered rAdV-ZW or PBS and not exposed to PEDV. Viral RNA excretion occurred in the other groups after PEDV infection; the virus shedding titer in the rAdV-ZW group was significantly lower than in rAdV-wild-type and PBS groups at 3 dpc (P < 0.05). Therefore, scFvs inhibited PEDV proliferation *in vivo* by blocking the PEDV N protein.

Viral infection elicits host immune responses, leading to changes in cytokine expression levels. Expression of the pro-inflammatory cytokines TNF-α, IL-6, IL-8, and IL-12 was up-regulated in PEDV-infected IPEC-J2 cells (61, 62), while PEDV infection increased TNF-α, IL-6, and IL-12 serum expression levels in piglets (63). TNF-α, IL-6, IL-8, and IL-12 serum expression levels in piglets on day 3 after PEDV challenge were significantly increased when compared with control IV and V groups (P < 0.05). After challenge, serum cytokine expression in piglets treated with rAdV-ZW was distinctly decreased when compared with group III piglets (P < 0.05), while rAdV-wild-type had no obvious effects on expression. Therefore, the N protein had important roles during proinflammatory cytokine production induced by PEDV, while scFvs effectively antagonized cytokine production.

PEDV mainly infects porcine small intestinal epithelial cells and proliferates in cells leading to atrophy and small intestinal villi shedding. Type III IFN has important roles in mucosal immunity which exhibits potent antiviral activity (64). Porcine IFN-λ1 and IFN-λ3 inhibit PEDV infection in IPEC-J2 cells (65, 66). To generate a cellular environment conducive to viral growth, PEDV has evolved several mechanisms to counteract or evade antiviral effects. Among PEDV proteins, 11, including nsp1, nsp3, nsp5, nsp8, nsp14, nsp15, nsp16, ORF3, E, M, and N suppressed IFN-λ1 promoter activities (67). Recent studies reported that the PEDV N protein antagonized IFN-λ3 production by blocking nuclear translocation of nuclear factor-κB (68). In our previous study, IFN-λ1 expression was up-regulated in PEDV-infected IPEC-J2 cells expressing all four scFvs against the PEDV N protein (36). As shown in **Supplementary Figure 2**, IFN-λ1 promoter activity was increased by intracellular scFv expression in a concentration-dependent manner. We also analyzed type III IFN serum expression levels; IFN-λ expression was dramatically downregulated after PEDV infection (P < 0.05). When piglets were administered rAdV-ZW before PEDV infection, IFN-λ expression was significantly upregulated (P < 0.05). These results were consistent with our previous study which showed the effects of all four scFvs against the PEDV N protein on type III IFN production in IPEC-J2 cells. Therefore, we hypothesize that our four scFvs expressed in the jejunum antagonized the negative regulatory effects of the PEDV N protein toward IFN-λ production by blocking the N protein *in vivo*.

In summary, four scFvs specifically targeting the PEDV N protein were cloned into adenovirus vectors and successfully expressed *in vitro* and *in vivo*. Recombinant adenoviruses were biosafe for piglets, and adenovirus vector-mediated scFvs effectively expressed in jejunum tissue were co-localized with the PEDV N protein in jejunal epithelial cells in PEDV-infected piglets. Further animal studies showed that a rAdV-scFv cocktail generated effective protective effects against PEDV infection. After scFv administration, IFN-λ serum expression levels in infected piglets were up-regulated, consistent with our previous study and providing new mechanistic insights on how the PEDV N protein antagonizes type III IFN expression. Our results provide encouraging antiviral potential of anti-N scFvs and a basis for developing scFv-based drugs to control PEDV-induced diarrhea in piglets. Additionally, the optimal level of recombinant adenovirus and the optimal time of PEDV challenge after oral administration of recombinant adenovirus are necessary to investigate to achieve optimal protection for piglets and the investigation is under the way.

## Data availability statement

The original contributions presented in the study are included in the article/**Supplementary Material**. Further inquiries can be directed to the corresponding authors.

## Ethics statement

The animal study was reviewed and approved by Animal Ethics Committee of Shanghai Jiao Tong University, School of Agriculture and Biology.

## Author contributions

FW, QZ, FZ, and JZ designed the research. JZ, QZ and ZY applied for funding. FW, EZ, ML, SM, and JG performed experiments. FW and QZ performed data analysis and manuscript writing. JZ supervised the project. All authors contributed to the article and approved the submitted version.
