# Supplementary material for: Adenovirus vector-mediated single chain variable fragments target the nucleocapsid protein of porcine epidemic diarrhea virus and protect against viral infection in piglets
Source: Front Immunol. 2023 Jan 24;14:1058327. doi: 10.3389/fimmu.2023.1058327 (PMC9902916; doi:10.3389/fimmu.2023.1058327)
Supplement: Supplementary file 1 [file Image_1.pdf]

Supplementary Material

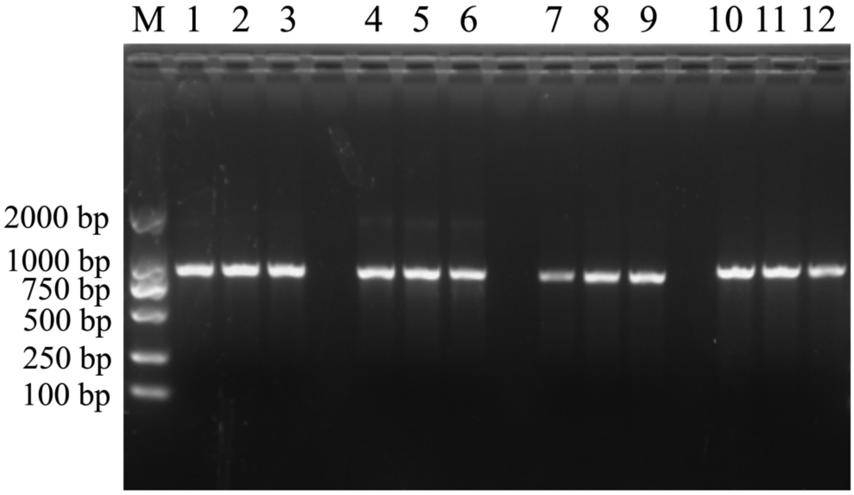

Supplementary Figure 1. PCR amplification of the four scFv genes. Lane M, 2,000-bp DNA ladder; lanes 1-3, PCR product of ZW1-16 gene; lanes 4-6, PCR product of ZW3-21 gene; lanes 7-9, PCR product of ZW1-41 gene; lanes 10-12, PCR product of ZW4-16 gene.

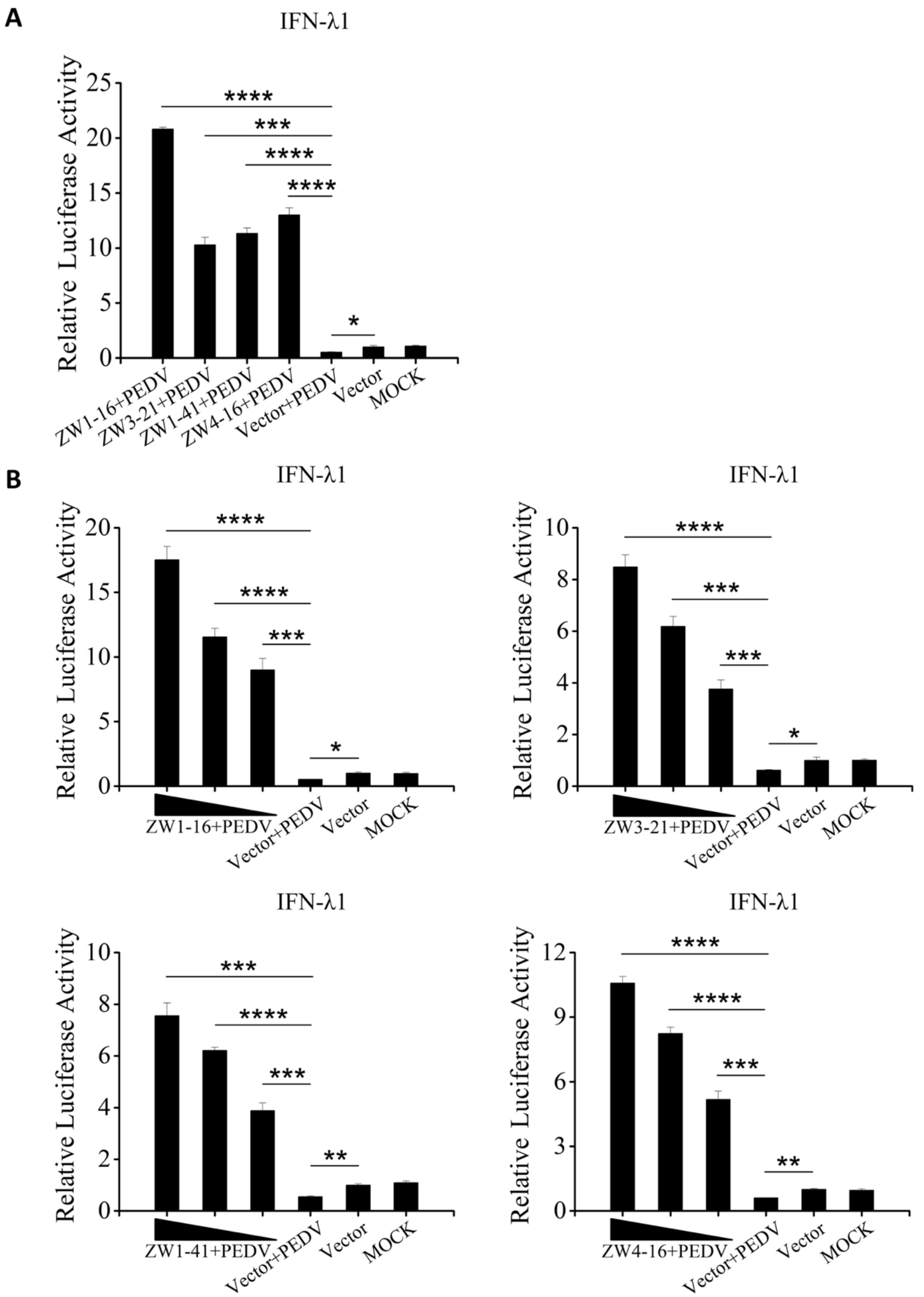

Supplementary Figure 2. Effect of the scFvs against PEDV N protein on promoter activation of IFN-λ1. **A)** IPEC-J2 cells were cotransfected with 1.2 μg of recombinant plasmid pCMV-HA-ZW1-16, pCMV-HA-ZW3-21, pCMV-HA-ZW1-41, pCMV-HA-ZW4-16, or pCMV-HA, 0.4 μg of recombinant plasmid pGL3-Basic-IFN-λ1-P containing IFN-λ1 promoter together with 0.04 μg of the plasmid pRL-

TK. **B)** Analysis of IFN-λ1 promoter activities under different concentrations (1.2, 0.8, or 0.4μg) of pCMV-HA-ZW1-16, pCMV-HA-ZW3-21, pCMV-HA-ZW1-41 and pCMV-HA-ZW4-16, respectively. The cells were infected with PEDV at 24 hpt. Luciferase assays were performed at 12 hpi. Untransfected uninfected IPEC-J2 cells were used as the MOCK control. The relative firefly luciferase activity was normalized to the *Renilla reniformis* luciferase activity, and the untreated empty vector control value was set to 1. Values are the means ± standard errors of the means (mean ± SEM) of three independent experiments. * $p < 0.05$, ** $p < 0.01$, *** $p < 0.001$, **** $p < 0.0001$.
